# 1,3-Bis[(−)-(*S*)-(1-phenyl­eth­yl)imino­meth­yl]benzene

**DOI:** 10.1107/S1600536811021556

**Published:** 2011-06-11

**Authors:** Tania García, Sylvain Bernès, Marcos Flores-Alamo, Guadalupe Hernández, René Gutiérrez

**Affiliations:** a DEP Facultad de Ciencias Químicas, UANL, Guerrero y Progreso S/N, Col. Treviño, 64570 Monterrey, N.L., Mexico; b Facultad de Química, Universidad Nacional Autónoma de México, México D.F. 04510, Mexico; c Laboratorio de Síntesis de Complejos, Facultad de Ciencias Químicas, Universidad Autónoma de Puebla, A.P. 1067, 72001 Puebla, Pue., Mexico

## Abstract

The title compound, C_24_H_24_N_2_, is an enanti­omerically pure bis-aldimine, which displays twofold crystallographic symmetry, with two C atoms of the central benzene ring lying on the symmetry axis. The imine group is slightly twisted from the benzene core, with a dihedral angle of 12.72 (16)° between the benzene ring and the C=N—C^*^ plane. The terminal phenyl rings make an angle of 66.44 (4)° and are oriented in opposite directions with respect to the benzene ring. In the crystal, mol­ecules inter­act weakly through a C—H⋯π inter­action involving the phenyl rings, and form chains along the 2_1_ screw-axis in the [100] direction.

## Related literature

For the structure of the analogous mol­ecule with naphthyl in place of phenyl, see: Espinosa Leija *et al.* (2009 ▶). For the structure of the isoformular mol­ecule with a 1,4-disubstituted benzene ring, see: García *et al.* (2010 ▶). For the Pd(II) and Pt(II) coordination complexes formed using the title ligand, see: Fossey *et al.* (2007 ▶). For background to the synthesis carried out in solvent-free conditions, see: Tanaka & Toda (2000 ▶); Jeon *et al.* (2005 ▶).
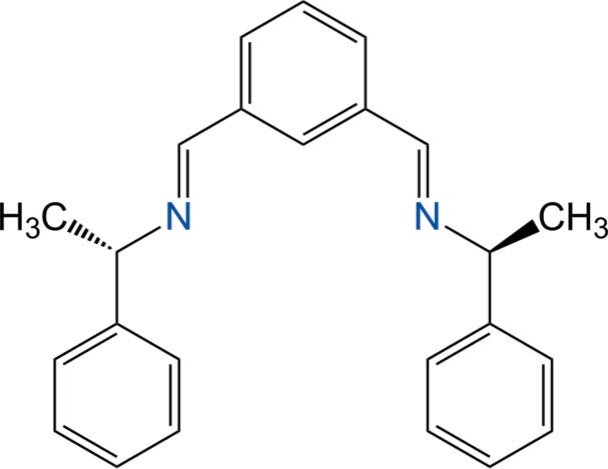


## Experimental

### 

#### Crystal data

C_24_H_24_N_2_
*M* *_r_* = 340.45
Orthorhombic, 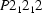

*a* = 21.1309 (7) Å
*b* = 5.6572 (2) Å
*c* = 8.2290 (3) Å
*V* = 983.71 (6) Å^3^
*Z* = 2
Mo *K*α radiation
μ = 0.07 mm^−1^
*T* = 130 K
0.33 × 0.26 × 0.14 mm

#### Data collection

Oxford Diffraction Xcalibur Atlas Gemini diffractometer
Absorption correction: analytical [*CrysAlis PRO* (Oxford Diffraction, 2009 ▶) based on expressions derived by Clark & Reid (1995 ▶)] *T*_min_ = 0.980, *T*_max_ = 0.991
6971 measured reflections
1161 independent reflections
1027 reflections with *I* > 2σ(*I*)
*R*_int_ = 0.025

#### Refinement

*R*[*F* ^2^ > 2σ(*F* ^2^)] = 0.029
*wR*(*F* ^2^) = 0.073
*S* = 1.06
1161 reflections
154 parameters
Only H-atom coordinates refined
Δρ_max_ = 0.09 e Å^−3^
Δρ_min_ = −0.17 e Å^−3^

### 

Data collection: *CrysAlis CCD* (Oxford Diffraction, 2009 ▶); cell refinement: *CrysAlis RED* (Oxford Diffraction, 2009 ▶); data reduction: *CrysAlis RED*; program(s) used to solve structure: *SHELXS97* (Sheldrick, 2008 ▶); program(s) used to refine structure: *SHELXL97* (Sheldrick, 2008 ▶); molecular graphics: *SHELXTL* (Sheldrick, 2008 ▶) and *Mercury* (Macrae *et al.*, 2006 ▶); software used to prepare material for publication: *SHELXTL*.

## Supplementary Material

Crystal structure: contains datablock(s) I, global. DOI: 10.1107/S1600536811021556/lr2012sup1.cif

Structure factors: contains datablock(s) I. DOI: 10.1107/S1600536811021556/lr2012Isup2.hkl

Supplementary material file. DOI: 10.1107/S1600536811021556/lr2012Isup3.mol

Additional supplementary materials:  crystallographic information; 3D view; checkCIF report

## Figures and Tables

**Table 1:** Hydrogen-bond geometry (Å, °) *Cg* is the centroid of the C1–C6 ring.

*D*—H⋯*A*	*D*—H	H⋯*A*	*D*⋯*A*	*D*—H⋯*A*
C3—H3⋯*Cg*^i^	0.92 (2)	2.97 (2)	3.7265 (18)	140.7 (18)

Symmetry code: (i) 
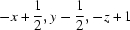
.

**Fig. 1.**
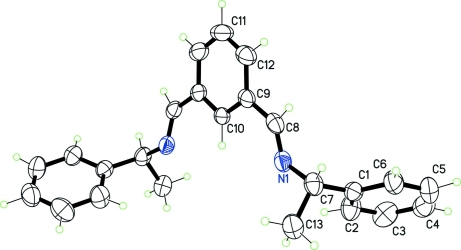
The title molecule with displacement ellipsoids for non-H atoms shown at the 60% probability level. Non-labeled atoms are generated by symmetry operation -x, 1 - y, z.

**Fig. 2.**
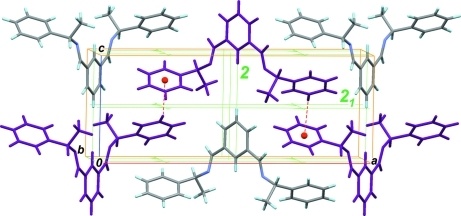
A part of the crystal structure of the title compound. The purple molecules are related by the 21 symmetry along the a axis, and dashed lines represent C—H···π contacts.

## supplementary crystallographic information

### Comment

The title compound was synthesized in an almost quantitative yield, using a one-step solvent-free route. Such procedures are becoming primordial in organic synthetic methods, in order to minimize the quantity of toxic waste and byproducts and to decrease the amount of solvents in the reaction media and/or during the following workups. Indeed, solvent-free reactions or solid-state reactions have been particularly developed these last years. (Jeon *et al.*, 2005; Tanaka & Toda, 2000).

The molecular structure of the title compound is as expected. The imine groups N1═C8 are found in the *E* configuration, which is known to be more stable than *Z*. The molecule is placed on a crystallographic twofold axis, passing through benzene atoms C10 and C11 (Fig. 1). Imine groups are not fully conjugated with the benzene core: the dihedral angle between C7—N1═ C8 and benzene mean-planes is 12.72 (16)°. The benzene ring makes an angle of 69.35 (5)° with the phenyl group, and terminal phenyl rings make an angle of 66.44 (4)°. Although the analogous bis-imine bearing a naphthyl group in place of phenyl crystallizes in the same space group, *P*2_1_2_1_2, and with identical molecular symmetry, it is stabilized in a different conformation compared to the title molecule. For instance, imine groups are almost perfectly conjugated with the benzene ring (dihedral angle between benzene and C^*^—N═C planes less than 0.6°; Espinosa Leija *et al.*, 2009). The title molecule and the naphthyl analogue are also differentiated by the fact that the latter crystallized with lattice solvent, CH_2_Cl_2_. The title molecule also shows a different conformation to that of the isoformular compound with a central 1,4-disubsituted benzene ring (García *et al.*, 2010): in that case, the molecule crystallizes in *P*2_1_2_1_2_1_ and is placed in general position (*C*_1_ point group).

The crystal structure (Fig. 2) features chains of molecules placed along the 2_1_ screw-axis in the [100] direction, which interact trough rather weak C3—H3···π contacts involving phenyl groups C1···C6. The H3···π separation is 2.97 (2) Å, and the C3—H3···π angle 140.7 (18)°.

Interestingly, (Fossey *et al.* 2007) reported on the synthesis of chiral bis-aldimine NCN–pincer complexes, where the NCN ligand is derived from the title compound by deprotonation at C10. These authors probed the catalytic activity of Pd(II) and Pt(II) complexes, where the ancillary ligand is an halide ion, Br^-^ or Cl^-^, for Pd and Pt complexes, respectively. The studied reaction, a classical Michael addition between methyl 2-cyanopropanoate and methyl vinyl ketone, showed that addition was not stereocontrolled. This poor selectivity was related to conformational flexibility of the chiral phenylethyl moiety of the ligand. Indeed, that point is confirmed by our structure, since a poor overlay is observed for this part of the molecule, when attempting to fit the title molecule and the main ligand in the complexes. However, differences in point symmetry also deserve to be considered regarding the catalytic activity: the title molecule belongs to *C*_2_ point-group, while complexes prepared by Fossey *et al.* crystallize in space group *P*2_1_2_1_2_1_, the whole complexes being placed in general positions. The complexes used for the Michael addition thus actually displayed the non-crystallographic *C*_2_ symmetry.

### Experimental

Under solvent-free conditions, (*S*)-(–)-1-phenylethylamine (0.45 g, 3.72 mmol) and benzene-1,3-dicarboxaldehyde (0.25 g, 1.86 mmol) in a 2:1 molar ratio were mixed at room temperature, obtaining a white solid. The crude was recrystallized twice from CH_2_Cl_2_, affording colorless crystals of the title compound. Yield: 92%; m.p. 80–82 °C. Analysis: [α]^25^_D_ = -71.4 (*c*=1, CHCl_3_). FT—IR (KBr): 1645 cm^-1^ (C=N). ^1^H-NMR (400 MHz, CDCl_3_/TMS) δ = 1.58 (d, 6H, CHC*H_3_*), 4.53 (q, 2H, C*H*), 7.22–8.11 (m, 14H, Ar—C*H*), 8.36 (s, 2H, *H*C=N). ^13^C-NMR (100 MHz, CDCl_3_/TMS) δ = 24.7 (C*C*H_3_), 69.6 (*C*HCH_3_), 126.5 (Ar), 126.8 (Ar), 128.2 (Ar), 128.3 (Ar), 128.7 (Ar), 130.0 (Ar), 136.6 (Ar), 144.8 (Ar), 158.9 (H*C*=N). MS—EI: *m*/*z*= 340 (*M*^+^).

### Refinement

All H atoms were found in a difference map and refined with free coordinates and isotropic displacement parameters fixed to *U*_iso_ = 1.2*U*_eq_(carrier C atom). C—H bond lengths are in the range 0.92 (2)–1.023 (18) Å. The absolute configuration at C7 was assigned from the known configuration of the chiral amine used as starting material, and measured Friedel pairs (775) were merged.

### Crystal data

**Table:**

C_24_H_24_N_2_	*D*_x_ = 1.149 Mg m^−^^3^
*M**_r_* = 340.45	Melting point: 353 K
Orthorhombic, *P*2_1_2_1_2	Mo *K*α radiation, λ = 0.71073 Å
Hall symbol: P 2 2ab	Cell parameters from 4133 reflections
*a* = 21.1309 (7) Å	θ = 3.6–26.0°
*b* = 5.6572 (2) Å	µ = 0.07 mm^−^^1^
*c* = 8.2290 (3) Å	*T* = 130 K
*V* = 983.71 (6) Å^3^	Prism, colourless
*Z* = 2	0.33 × 0.26 × 0.14 mm
*F*(000) = 364	

### Data collection

**Table:**

Oxford Diffraction Xcalibur Atlas Gemini diffractometer	1161 independent reflections
Radiation source: Enhance (Mo) X-ray Source	1027 reflections with *I* > 2σ(*I*)
graphite	*R*_int_ = 0.025
Detector resolution: 10.4685 pixels mm^-1^	θ_max_ = 26.0°, θ_min_ = 3.7°
ω scans	*h* = −26→24
Absorption correction: analytical [*CrysAlis PRO* (Oxford Diffraction, 2009) based on expressions derived by Clark & Reid (1995)]	*k* = −6→6
*T*_min_ = 0.980, *T*_max_ = 0.991	*l* = −10→9
6971 measured reflections	

### Refinement

**Table:**

Refinement on *F*^2^	Primary atom site location: structure-invariant direct methods
Least-squares matrix: full	Secondary atom site location: difference Fourier map
*R*[*F*^2^ > 2σ(*F*^2^)] = 0.029	Hydrogen site location: inferred from neighbouring sites
*wR*(*F*^2^) = 0.073	Only H-atom coordinates refined
*S* = 1.06	*w* = 1/[σ^2^(*F*_o_^2^) + (0.0497*P*)^2^ + 0.0095*P*] where *P* = (*F*_o_^2^ + 2*F*_c_^2^)/3
1161 reflections	(Δ/σ)_max_ < 0.001
154 parameters	Δρ_max_ = 0.09 e Å^−^^3^
0 restraints	Δρ_min_ = −0.17 e Å^−^^3^
0 constraints	

### Fractional atomic coordinates and isotropic or equivalent isotropic displacement parameters (Å^2^)

**Table:**

	*x*	*y*	*z*	*U*_iso_*/*U*_eq_	
N1	0.08171 (5)	0.8507 (2)	0.09804 (15)	0.0334 (3)	
C1	0.18464 (7)	0.9616 (3)	0.20948 (18)	0.0320 (3)	
C2	0.19358 (8)	0.7590 (3)	0.3015 (2)	0.0444 (4)	
H2	0.1570 (9)	0.669 (3)	0.330 (2)	0.053*	
C3	0.25402 (9)	0.6851 (3)	0.3437 (2)	0.0517 (5)	
H3	0.2581 (9)	0.549 (4)	0.405 (3)	0.062*	
C4	0.30581 (8)	0.8153 (4)	0.2952 (2)	0.0504 (5)	
H4	0.3488 (10)	0.765 (4)	0.326 (2)	0.060*	
C5	0.29724 (8)	1.0147 (4)	0.2039 (2)	0.0543 (5)	
H5	0.3325 (10)	1.099 (4)	0.164 (3)	0.065*	
C6	0.23701 (8)	1.0880 (3)	0.1605 (2)	0.0420 (4)	
H6	0.2300 (8)	1.226 (4)	0.100 (2)	0.050*	
C7	0.11876 (7)	1.0481 (3)	0.16569 (19)	0.0346 (4)	
H7	0.1234 (8)	1.176 (3)	0.0885 (19)	0.042*	
C8	0.06895 (6)	0.8605 (3)	−0.05179 (18)	0.0320 (4)	
H8	0.0837 (7)	0.997 (3)	−0.1224 (19)	0.038*	
C9	0.03271 (6)	0.6758 (3)	−0.13695 (17)	0.0313 (3)	
C10	0.0000	0.5000	−0.0535 (2)	0.0299 (5)	
H10	0.0000	0.5000	0.068 (3)	0.036*	
C11	0.0000	0.5000	−0.3906 (3)	0.0441 (6)	
H11	0.0000	0.5000	−0.506 (3)	0.053*	
C12	0.03173 (7)	0.6752 (3)	−0.30697 (18)	0.0398 (4)	
H12	0.0545 (8)	0.797 (3)	−0.363 (2)	0.048*	
C13	0.08354 (8)	1.1472 (3)	0.3116 (2)	0.0427 (4)	
H13A	0.1078 (8)	1.284 (4)	0.365 (2)	0.051*	
H13B	0.0793 (9)	1.021 (4)	0.393 (2)	0.051*	
H13C	0.0397 (9)	1.208 (3)	0.279 (2)	0.051*	

### Atomic displacement parameters (Å^2^)

**Table:**

	*U*^11^	*U*^22^	*U*^33^	*U*^12^	*U*^13^	*U*^23^
N1	0.0258 (6)	0.0373 (7)	0.0370 (7)	−0.0034 (5)	0.0025 (5)	0.0064 (6)
C1	0.0324 (8)	0.0313 (7)	0.0325 (7)	−0.0046 (6)	0.0019 (6)	−0.0027 (7)
C2	0.0393 (9)	0.0360 (8)	0.0580 (11)	−0.0068 (7)	−0.0027 (8)	0.0085 (8)
C3	0.0564 (10)	0.0411 (10)	0.0578 (11)	0.0074 (9)	−0.0127 (9)	0.0024 (9)
C4	0.0371 (9)	0.0653 (12)	0.0489 (10)	0.0111 (9)	−0.0053 (8)	−0.0156 (10)
C5	0.0322 (9)	0.0736 (13)	0.0571 (11)	−0.0111 (9)	0.0038 (8)	0.0015 (11)
C6	0.0361 (9)	0.0460 (10)	0.0440 (9)	−0.0079 (7)	0.0035 (7)	0.0063 (8)
C7	0.0322 (8)	0.0310 (7)	0.0407 (8)	−0.0052 (7)	0.0028 (7)	0.0081 (7)
C8	0.0252 (7)	0.0355 (8)	0.0351 (8)	0.0052 (6)	0.0063 (6)	0.0079 (7)
C9	0.0233 (6)	0.0406 (8)	0.0301 (7)	0.0105 (7)	0.0020 (6)	0.0036 (7)
C10	0.0216 (9)	0.0413 (12)	0.0268 (10)	0.0084 (9)	0.000	0.000
C11	0.0454 (13)	0.0633 (16)	0.0236 (11)	0.0175 (12)	0.000	0.000
C12	0.0347 (8)	0.0520 (10)	0.0326 (8)	0.0119 (8)	0.0048 (7)	0.0090 (8)
C13	0.0360 (8)	0.0424 (9)	0.0496 (10)	−0.0023 (8)	0.0046 (8)	−0.0012 (9)

### Geometric parameters (Å, °)

**Table:**

N1—C8	1.2633 (19)	C7—H7	0.967 (17)
N1—C7	1.4727 (19)	C8—C9	1.473 (2)
C1—C6	1.378 (2)	C8—H8	1.014 (18)
C1—C2	1.387 (2)	C9—C10	1.3920 (17)
C1—C7	1.519 (2)	C9—C12	1.399 (2)
C2—C3	1.388 (2)	C10—C9^i^	1.3920 (17)
C2—H2	0.96 (2)	C10—H10	1.00 (2)
C3—C4	1.378 (3)	C11—C12^i^	1.380 (2)
C3—H3	0.92 (2)	C11—C12	1.380 (2)
C4—C5	1.367 (3)	C11—H11	0.95 (2)
C4—H4	0.98 (2)	C12—H12	0.959 (19)
C5—C6	1.385 (2)	C13—H13A	1.02 (2)
C5—H5	0.94 (2)	C13—H13B	0.98 (2)
C6—H6	0.94 (2)	C13—H13C	1.023 (18)
C7—C13	1.520 (2)		
			
C8—N1—C7	116.69 (13)	C1—C7—H7	107.7 (10)
C6—C1—C2	118.64 (15)	C13—C7—H7	107.0 (9)
C6—C1—C7	119.96 (14)	N1—C8—C9	122.96 (14)
C2—C1—C7	121.38 (13)	N1—C8—H8	121.8 (9)
C1—C2—C3	120.71 (17)	C9—C8—H8	115.3 (9)
C1—C2—H2	117.7 (12)	C10—C9—C12	118.95 (16)
C3—C2—H2	121.5 (11)	C10—C9—C8	122.03 (13)
C4—C3—C2	119.84 (17)	C12—C9—C8	119.01 (15)
C4—C3—H3	121.9 (12)	C9—C10—C9^i^	120.90 (18)
C2—C3—H3	118.2 (12)	C9—C10—H10	119.55 (9)
C5—C4—C3	119.65 (16)	C9^i^—C10—H10	119.55 (9)
C5—C4—H4	120.0 (12)	C12^i^—C11—C12	120.2 (2)
C3—C4—H4	120.4 (12)	C12^i^—C11—H11	119.90 (10)
C4—C5—C6	120.69 (17)	C12—C11—H11	119.90 (10)
C4—C5—H5	120.4 (12)	C11—C12—C9	120.48 (17)
C6—C5—H5	118.8 (12)	C11—C12—H12	121.4 (11)
C1—C6—C5	120.45 (16)	C9—C12—H12	118.1 (11)
C1—C6—H6	117.4 (11)	C7—C13—H13A	111.8 (10)
C5—C6—H6	122.1 (11)	C7—C13—H13B	108.4 (11)
N1—C7—C1	109.44 (12)	H13A—C13—H13B	107.4 (14)
N1—C7—C13	108.55 (12)	C7—C13—H13C	111.2 (9)
C1—C7—C13	112.35 (13)	H13A—C13—H13C	108.1 (13)
N1—C7—H7	111.8 (10)	H13B—C13—H13C	109.8 (15)
			
C6—C1—C2—C3	0.1 (2)	C2—C1—C7—N1	−49.69 (19)
C7—C1—C2—C3	−178.53 (16)	C6—C1—C7—C13	−107.63 (17)
C1—C2—C3—C4	0.6 (3)	C2—C1—C7—C13	70.97 (19)
C2—C3—C4—C5	−0.9 (3)	C7—N1—C8—C9	179.55 (12)
C3—C4—C5—C6	0.4 (3)	N1—C8—C9—C10	12.2 (2)
C2—C1—C6—C5	−0.6 (2)	N1—C8—C9—C12	−167.04 (14)
C7—C1—C6—C5	178.06 (16)	C12—C9—C10—C9^i^	0.71 (10)
C4—C5—C6—C1	0.3 (3)	C8—C9—C10—C9^i^	−178.53 (14)
C8—N1—C7—C1	−110.27 (15)	C12^i^—C11—C12—C9	0.73 (10)
C8—N1—C7—C13	126.80 (15)	C10—C9—C12—C11	−1.4 (2)
C6—C1—C7—N1	131.72 (14)	C8—C9—C12—C11	177.83 (10)

Symmetry codes: (i) −*x*, −*y*+1, *z*.

### Hydrogen-bond geometry (Å, °)

**Table:**

Cg is the centroid of the C1–C6 ring.

**Table:**

*D*—H···*A*	*D*—H	H···*A*	*D*···*A*	*D*—H···*A*
C3—H3···Cg^ii^	0.92 (2)	2.97 (2)	3.7265 (18)	140.7 (18)

Symmetry codes: (ii) −*x*+1/2, *y*−1/2, −*z*+1.
